# Supplementary material for: Bedrock geochemistry influences vegetation growth by regulating the regolith water holding capacity
Source: Nat Commun. 2020 May 13;11:2392. doi: 10.1038/s41467-020-16156-1 (PMC7220924; doi:10.1038/s41467-020-16156-1)
Supplement: Supplementary file 1 — Supplementary tables and figures [file 41467_2020_16156_MOESM1_ESM.pdf]

Supporting information for Bedrock geochemistry influences vegetation growth by regulating the regolith water holding capacity by Jiang et al

Supplementary Table 1. Pearson correlation coefficients of all candidate variables for model selection considering NPP and RWLR predictions. R values greater than |0.70| are in bold.

	RWLR	MAT	MAP	PDSI	Soil N	SD
RWLR	1.00	-0.60	-0.15	0.01	0.02	0.08
MAT	-0.60	1.00	0.12	0.14	-0.12	-0.015
MAP	-0.15	0.12	1.00	-0.23	0.21	0.21
PDSI	0.01	0.14	-0.23	1.00	0.28	-0.18
Soil N	0.02	-0.12	0.21	0.28	1.00	0.10
SD	0.08	-0.015	0.22	-0.18	0.10	1.00

	BR_{Si}	BR_{Ca}	BR_{Mg}	BR_{Al}
BR_{Si}	1.00	**-0.80**	-0.34	**0.81**
BR_{Ca}	**-0.80**	1.00	0.14	-0.69
BR_{Mg}	-0.34	0.14	1.00	-0.23
BR_{Fe}	0.58	-0.42	0.00	**0.79**
BR_{Al}	**0.81**	-0.69	-0.23	1.00

Supplementary Table 2. Best fitting model subsets for NPP and RWLR. The difference in AIC between the best fitting model and the present model (ΔAIC) and Akaike weights (w_i) were obtained from applying an information theoretic approach. Models were ranked based on w_i, which measures the relative goodness of fit of each model considering the overall model complexity (through a model-complexity penalization factor). Unshaded cells indicate the variables were not included in the model.

Table S2a. Best fitting models subset for NPP

<table>
<tr><th>RWLR</th><th>Soil N</th><th>SD</th><th>MAP</th><th>MAT</th><th>PDSI</th><th>ΔAIC_C</th><th>w_i</th></tr>
<tr><td bgcolor="yellow"></td><td></td><td></td><td></td><td></td><td></td><td>0.00</td><td>0.20</td></tr>
<tr><td bgcolor="yellow"></td><td bgcolor="yellow"></td><td></td><td></td><td></td><td></td><td>0.16</td><td>0.19</td></tr>
<tr><td bgcolor="yellow"></td><td></td><td bgcolor="yellow"></td><td></td><td></td><td></td><td>1.82</td><td>0.08</td></tr>
<tr><td bgcolor="yellow"></td><td></td><td></td><td bgcolor="yellow"></td><td></td><td></td><td>2.07</td><td>0.07</td></tr>
<tr><td bgcolor="yellow"></td><td bgcolor="yellow"></td><td bgcolor="yellow"></td><td></td><td></td><td></td><td>2.53</td><td>0.06</td></tr>
<tr><td bgcolor="yellow"></td><td></td><td></td><td></td><td bgcolor="yellow"></td><td></td><td>2.60</td><td>0.05</td></tr>
<tr><td bgcolor="yellow"></td><td></td><td></td><td></td><td></td><td bgcolor="yellow"></td><td>2.72</td><td>0.05</td></tr>
<tr><td bgcolor="yellow"></td><td bgcolor="yellow"></td><td></td><td bgcolor="yellow"></td><td></td><td></td><td>3.07</td><td>0.04</td></tr>
<tr><td bgcolor="yellow"></td><td bgcolor="yellow"></td><td></td><td></td><td bgcolor="yellow"></td><td></td><td>3.30</td><td>0.04</td></tr>
<tr><td bgcolor="yellow"></td><td bgcolor="yellow"></td><td></td><td></td><td></td><td bgcolor="yellow"></td><td>3.46</td><td>0.04</td></tr>
<tr><td bgcolor="yellow"></td><td></td><td bgcolor="yellow"></td><td bgcolor="yellow"></td><td></td><td></td><td>4.60</td><td>0.02</td></tr>
<tr><td bgcolor="yellow"></td><td></td><td bgcolor="yellow"></td><td></td><td></td><td bgcolor="yellow"></td><td>4.62</td><td>0.02</td></tr>
</table>

						4.69	0.02
						4.84	0.02
						4.96	0.02
						5.54	0.01
						6.02	0.01
						6.06	0.01
						6.18	0.01

Table S2b. Best fitting models subset (including BR_{Si}) for the RWLR

BR_{Si}	BR_{Mg}	BR_{Al}	BR_{Fe}	ΔAIC_C	w_i
				0.00	0.46
				2.37	0.14
				2.38	0.14
				2.76	0.12
				5.33	0.03
				5.52	0.03
				5.68	0.03

Table S2c. Best fitting models subset (including BR_{Ca}) for the RWLR

BR_{Ca}	BR_{Mg}	BR_{Al}	BR_{Fe}	ΔAIC_C	w_i
				0.00	0.36
				0.34	0.30
				2.91	0.08
				2.95	0.08
				3.62	0.06
				3.64	0.06
				6.20	0.02

Supplementary Table 3 General features of the selected karst areas

**Location**	**Area (km^2)**	**MAT (℃)**	**AP (mm)**	**Main vegetation types**
West of Canada	10.75×10^4	0.1	820	Grassland, Cropland
Mid-Canada	15.69×10^4	1.0	502	Shrubland
Edge of Hudson bay, Canada	42.25×10^4	-3.4	565	Shrubland, Grassland
Texas, USA	12.25×10^4	18.5	578	Grassland
Florida, USA	22.25×10^4	20.3	1314	Mangrove
East of USA	85.75×10^4	10.2	1044	Forest, Grassland
France and Spain	38.00×10^4	10.9	769	Grassland, Forest
Balkan peninsula	25.5×10^4	9.6	1045	Grassland, Forest
Iran	8.50×10^4	15.1	320	Shrubland
Turkey	9.75×10^4	12.2	591	Shrubland, Grassland
Tibet, China	40.25×10^4	-3.9	293	Grassland
Russia	69.25×10^4	-6.5	390	Grassland, Forest

Supplementary Figure 1 Distribution of the a) lengths and b) months of the dry spells that occurred in the study region.

Supplementary Figure 2 Box-plots comparing carbonate and non-carbonate critical zones units (CZUs) as a function of climate, regolith properties and bedrock geochemistry. T-test was used to assess significant differences between carbonate and non-carbonate CZU. *P*-values less than 0.05 are in bold. Bars represent means for each treatment and error bars are 95% confidence intervals of the mean.

Supplementary Figure 3 Illustration diagram of the critical zone unit (CZU). A CZU is an area with 20-km radius and a meteorological station as its center. The area extent of each CZU based on the spatial representativeness of meteorological station data[31]. The square represents the meteorological station, with the different shades of grey representing the different rock types, and triangles representing the selected soil and bedrock sampling sites.

Supplementary Figure 4 Illustration diagram of the method used to calculated the regolith water loss rate. Day_1 is the date when a rainfall event occurred and consist of the date of $TVDI_1$. Dry spell is the period with five or more consecutive days with zero rain, starting with the day after Day_1. $TVDI_i$ is a time series indicating the variation in the surface water deficit during the dry spell. Variations in the TVDI were calculated along the temporal gradient.

Figure S1

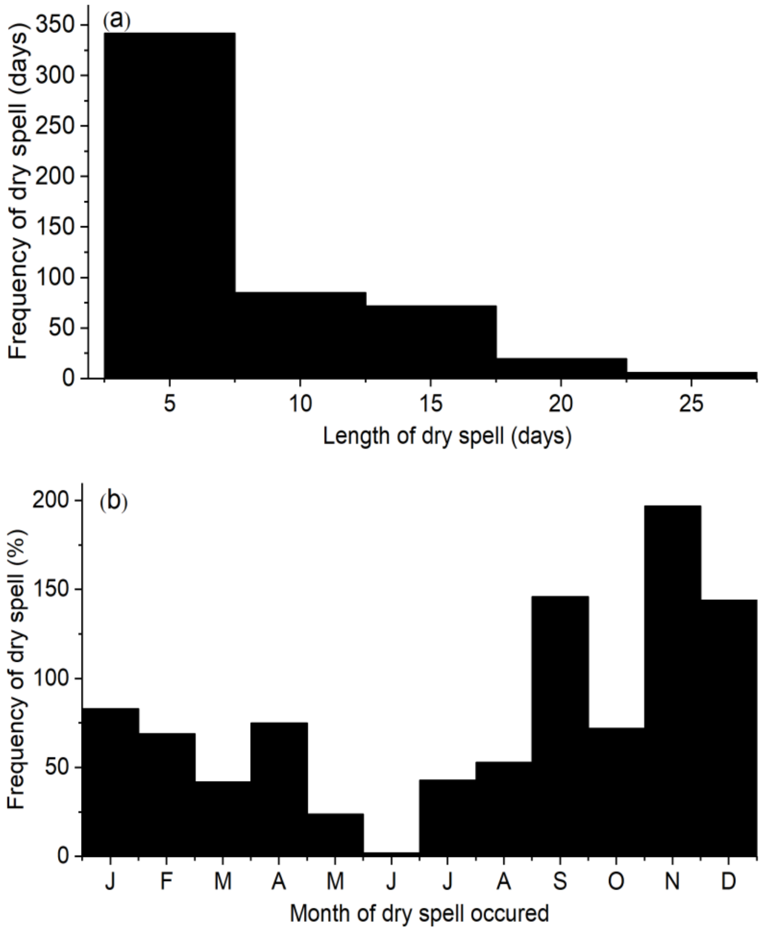
(a)
350
300
250
200
150
100
50
0
Frequency of dry spell (days)
5
10
15
20
25
Length of dry spell (days)
(b)
200
150
100
50
0
Frequency of dry spell (%)
J
F
M
A
M
J
J
A
S
O
N
D
Month of dry spell occured

Figure S2

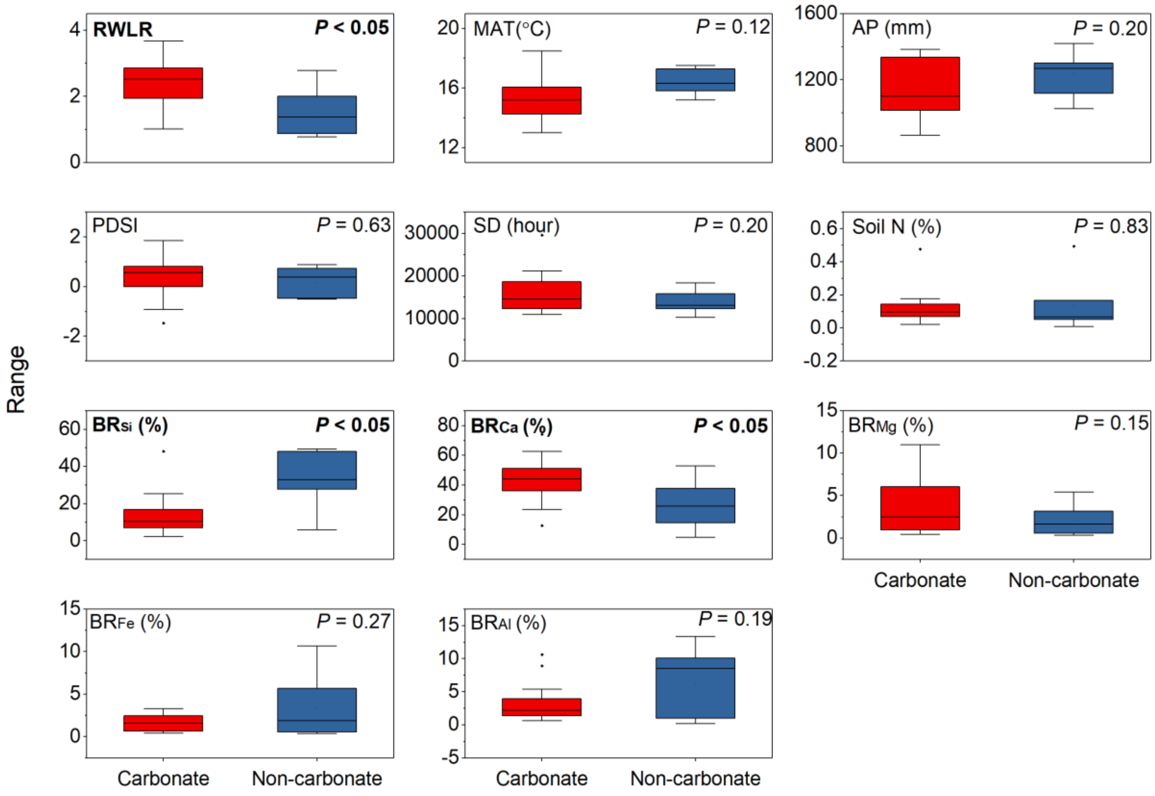

Range
RWLR
P < 0.05
MAT(°C)
P = 0.12
AP (mm)
P = 0.20
PDSI
P = 0.63
SD (hour)
P = 0.20
Soil N (%)
P = 0.83
BRSi (%)
P < 0.05
BRCa (%)
P < 0.05
BRMg (%)
P = 0.15
BRFe (%)
P = 0.27
BRAl (%)
P = 0.19
Carbonate
Non-carbonate

Figure S3

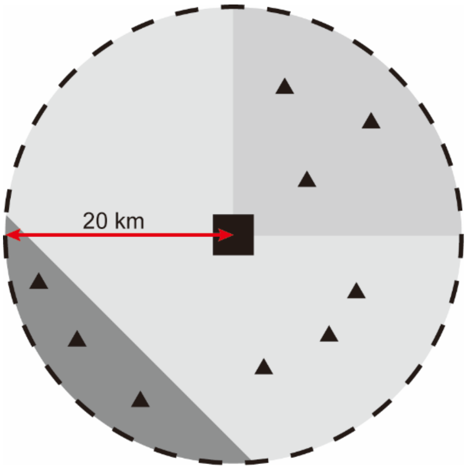

Figure S4

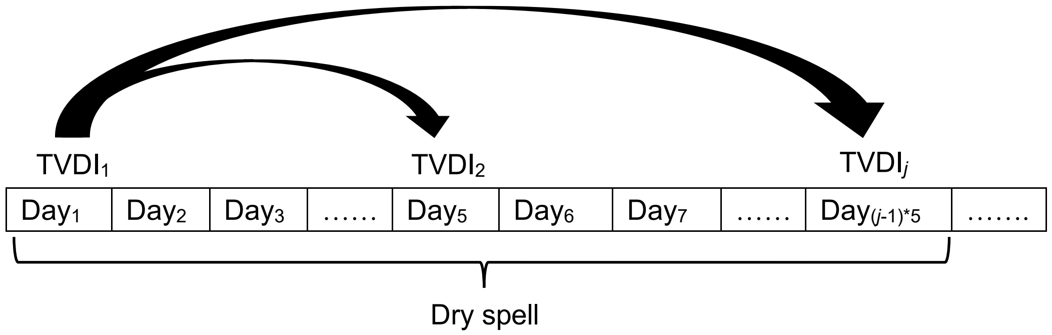

TVDI1
TVDI2
TVDIj
Day1
Day2
Day3
……
Day5
Day6
Day7
……
Day(j-1)*5
…….
Dry spell
